# Implications of ICU triage decisions on patient mortality: a cost-effectiveness analysis

**DOI:** 10.1186/cc10029

**Published:** 2011-02-09

**Authors:** David L Edbrooke, Cosetta Minelli, Gary H Mills, Gaetano Iapichino, Angelo Pezzi, Davide Corbella, Philip Jacobs, Anne Lippert, Joergen Wiis, Antonio Pesenti, Nicolo Patroniti, Romain Pirracchio, Didier Payen, Gabriel Gurman, Jan Bakker, Jozef Kesecioglu, Chris Hargreaves, Simon L Cohen, Mario Baras, Antonio Artigas, Charles L Sprung

**Affiliations:** 1 Faculty of Health and Wellbeing, Sheffield Hallam University, City Campus, Howard Street, Sheffield, S1 1WB, UK; 2 Medical and Economics Research Centre (MERCS) Sheffield, Royal Hallamshire Hospital, Sheffield Teaching Hospitals NHS Trust, Glossop Road, Sheffield S10 2JF, UK; 3 EURAC research, Viale Druso 1, 39100, Bolzano, Italy; 4 Critical Care, Anaesthesia and Operating Services, and Medical and Economics Research Centre (MERCS), Royal Hallamshire Hospital, Sheffield Teaching Hospitals NHS Trust, Glossop Road, Sheffield, S10 2JF, UK; 5 Istituto di Anestesiologia e Rianimazione, Universita degli Studi di Milano, UO Anestesia e Rianimazione, Polo Universitario San Paolo via Di Rudini, 20142, Milano, Italy; 6 Collaborations, Institute of Health Economics and Department of Medicine. University of Alberta, 1200 10405 Jasper Avenue, Edmonton, Alberta, T5J 3N4, Canada; 7 Department of Anaesthesia, Herlev University Hospital, Herlev Ringvej 75, 2730 Herlev, Copenhagen, Denmark; 8 San Gerardo Hospital, Università degli Studi Milano-Bicocca, Monza, Italy; 9 Department of Anaesthesia, Lariboisiere Hospital, Rue Ambroise Pare 2, 75475, Paris, France; 10 Soroka Medical Centre, Ben Gurion University of the Negev, Beer Sheva, 84141, Israel; 11 Isala Hospital, GM Zwolle, 8000, Netherlands; 12 University Medical Centre, Heidelberglaan 100, 3584CX, Utrecht, Netherlands; 13 Intensive Care Unit, Whittington Hospital NHS Trust, London N19 5NF, UK; 14 University College Hospital, Euston Rd, London, NW1 2BU, UK; 15 The Hebrew University, Haddasah School of Public Health, Haddasah Medical Centre, Jerusalem, 91120, Israel; 16 Critical Care Center Sabadell Hospital, CIBER Enfermedades Respiratorias, Corporacio Sanataria Parc Tauli, 08208, Sabadell, Spain; 17 Department of Anesthesiology and Critical Care, Hadassah Hebrew University Medical Centre, Jerusalem, 91120, Israel

## Abstract

**Introduction:**

Intensive care is generally regarded as expensive, and as a result beds are limited. This has raised serious questions about rationing when there are insufficient beds for all those referred. However, the evidence for the cost effectiveness of intensive care is weak and the work that does exist usually assumes that those who are not admitted do not survive, which is not always the case. Randomised studies of the effectiveness of intensive care are difficult to justify on ethical grounds; therefore, this observational study examined the cost effectiveness of ICU admission by comparing patients who were accepted into ICU after ICU triage to those who were not accepted, while attempting to adjust such comparison for confounding factors.

**Methods:**

This multi-centre observational cohort study involved 11 hospitals in 7 EU countries and was designed to assess the cost effectiveness of admission to intensive care after ICU triage. A total of 7,659 consecutive patients referred to the intensive care unit (ICU) were divided into those accepted for admission and those not accepted. The two groups were compared in terms of cost and mortality using multilevel regression models to account for differences across centres, and after adjusting for age, Karnofsky score and indication for ICU admission. The analyses were also stratified by categories of Simplified Acute Physiology Score (SAPS) II predicted mortality (< 5%, 5% to 40% and >40%). Cost effectiveness was evaluated as cost per life saved and cost per life-year saved.

**Results:**

Admission to ICU produced a relative reduction in mortality risk, expressed as odds ratio, of 0.70 (0.52 to 0.94) at 28 days. When stratified by predicted mortality, the odds ratio was 1.49 (0.79 to 2.81), 0.7 (0.51 to 0.97) and 0.55 (0.37 to 0.83) for <5%, 5% to 40% and >40% predicted mortality, respectively. Average cost per life saved for all patients was $103,771 (€82,358) and cost per life-year saved was $7,065 (€5,607). These figures decreased substantially for patients with predicted mortality higher than 40%, $60,046 (€47,656) and $4,088 (€3,244), respectively. Results were very similar when considering three-month mortality. Sensitivity analyses performed to assess the robustness of the results provided findings similar to the main analyses.

**Conclusions:**

Not only does ICU appear to produce an improvement in survival, but the cost per life saved falls for patients with greater severity of illness. This suggests that intensive care is similarly cost effective to other therapies that are generally regarded as essential.

## Introduction

Intensive care is generally regarded as expensive, with historical reports of the average cost per patient-day ranging from £858 to 1,185 in the UK [1]. Attempts to limit resources have raised the question of who gets admitted to ICU when there are insufficient beds for all referred patients [2,3]. This has raised serious ethical concerns worldwide due to the implications of ICU rationing [3,4] on patient outcomes [5,6]. However, the evidence for the cost effectiveness of intensive care is currently weak. Ideally a randomised study would answer this question. However, randomised studies of the cost effectiveness of intensive care are difficult to implement and justify ethically. Previous cost effectiveness evaluations [7-10] have generally assumed patients not admitted to intensive care die, which is not always the case [5,6]. If intensive care is to be measured against other forms of therapy, all of which are competing for scarce resources, some attempt at a cost-effectiveness analysis of admission to intensive care is urgently needed.

The present study is a cost-effectiveness analysis of ICU admission compared with ward care for patients referred for admission to ICU, in which clinical outcomes (28-day and 3-month mortality) and resource use were measured for both settings.

## Materials and methods

The present cost-effectiveness analysis is part of the Elderly in European Intensive Care Units (ELDICUS) project (QLK6-CT-2002-00251 EU FP5), a prospective multicentre cohort study investigating ICU triage decisions. The study received ethics committee approval from the institutional review board in all centres and the need for individual patient consent was waived.

Patients referred to the intensive care unit (ICU) were divided into those accepted for admission and those not accepted. The two groups were then compared in terms of mortality and cost as a whole and also in categories of Simplified Acute Physiology Score (SAPS) II predicted mortality.

### Study population

Consecutive adult patients (older than 18 years) referred for admission to ICU were recruited in 11 hospitals from 7 European Union or associated countries (Denmark, France, Israel, Italy, Netherlands, Spain and the UK), between September 2003 and March 2005. There was no upper limit on age. This was an observational study and no attempt was made to influence decisions to admit or not admit patients to ICU. Patients were referred to ICU by doctors in the emergency department, medical ward, surgical ward or operating room. Patients were excluded if they were referred to ICU for consultation only or from other ICUs as well as intermediate (high dependency) units in the same hospital. For patients with more than one ICU triage during the same hospital stay, only the first triage was considered in the present analyses.

### Effect of ICU admission on patient mortality

The effectiveness of ICU admission was evaluated by comparing the 28-day mortality for patients accepted in ICU with that of patients not accepted and treated in the ward. Three-month mortality was also evaluated as a secondary outcome. Although hospital mortality was also available, we did not use this outcome measure since this varies widely across centres and countries as a result of different policies for hospital discharge.

The case mix of patients accepted in ICU is likely to differ in terms of severity and prognosis from that of patients refused, and, therefore, analyses were adjusted for possible confounders.

Possible confounding variables were selected through backward stepwise procedure, and included; age; Karnofsky performance status (a marker of chronic health); and indication for referral to ICU (treatment or observation). Since the observed effect on mortality of ICU admission varied with the severity of illness (as measured by SAPS II), the analyses were stratified by categories of predicted mortality, calculated from the SAPS II score [11].

### Costs

Cost of ICU admission was evaluated by comparing the total hospital cost per patient for patients accepted to the ICU against the total cost for patients not accepted and treated on the ward. For patients accepted to ICU, the total cost was defined as the cost of the ICU stay, plus the ward stay cost after ICU discharge. The daily cost per patient for both ICU and ward was calculated for each participating hospital using a top-down approach, based on the cost-block method [1]. Although this approach has been developed for use in the ICU, it has also been used to calculate ward costs for consistency and in the absence of any other accepted method [12]. The cost-block method derives an average daily cost per patient from the total annual cost of the unit. The total annual cost is estimated as the sum of the main cost determinants, or "cost-blocks", which include; consumables (drugs, nutritional products, blood products and disposables); clinical support services defined as services essential to the ICU but not provided within the ICU (laboratory, radiology and physiotherapy); and ICU staff (doctors, junior and senior, and nurses). In the UK, cost-blocks have been shown to account for 85% of the total annual ICU cost, with the remaining 15%, described as "overheads", including maintenance costs of the hospital and ICU [1]. These overheads of 15% were thus added to the calculated costs. Data on cost-blocks were collected using the cost-block questionnaire [1]. In each hospital, the cost-block questionnaire was completed by the participating ICU and by one surgical and one medical ward. The annual ward cost was defined as the average of the costs of the two wards.

Daily costs per patient were calculated based on the annual cost and the number of beds, assuming an occupancy rate of 100%. In fact, a large part of the total cost of care is represented by staff cost, which does not normally depend on occupancy.

Between countries, costs were standardised to a common currency using Purchasing Power Parities (PPPs) rather than exchange rates, as the latter were designed to compare costs in financial markets which, unlike health service costs, change rapidly. The World Health Organisation (WHO) PPPs were used because they consider health costs, are reported in many countries and are frequently updated [13]. The numeraire currency is the International Dollar, a theoretical currency based on what can be bought in each country with the US dollar [14]. In practice the International Dollar corresponds to the US Dollar and so the International $ is simply referred to as $ in this study. To aid interpretation in Europe we have also converted our results to Euros using the Dollar to Euro conversion rate in place at the end of the study; that is, $1.26 is equivalent to €1.00. We have also applied this conversion rate to other papers quoted in our manuscript where a Euro figure was not available.

Cost estimates for the cost-effectiveness analyses were adjusted for the same covariates selected in the analysis of effectiveness (age, Karnofsky performance status, indication for referral to ICU), and cost-effectiveness analyses were stratified by severity of illness (predicted mortality based on SAPS II).

### Cost effectiveness of ICU admission

The cost effectiveness of ICU admission, compared with ward care, after ICU triage was evaluated using two measures; cost per life saved and cost per life-year saved. These were calculated at 28 days and 3 months after discharge for all admissions and represent the cost over and above ward care in relation to the benefit accrued.

#### Cost per life saved

The cost per life saved, or incremental cost-effectiveness ratio, is the difference in cost divided by the difference in mortality rates (absolute risk reduction), with the latter being calculated from the odds ratio derived from the adjusted analyses (see Additional file 1).

#### Cost per life-year saved

The calculation of a cost per life-year saved requires an estimate of the life expectancy for survivors (see Additional file 1), information which was obtained from published data. Life expectancy of ICU survivors differs from that of the general population only for the first two [15,16] to four [17,18] years after hospital discharge. Thus, a hypothetical value of life expectancy was assigned to each patient using general population estimates specific for age, gender and country, after accounting for excess mortality in the first four years after hospital discharge (see Additional file 1). Figures on life expectancy in the general population were obtained from EUROSTAT [19] for European countries, and from the U.S Census Bureau - International Data Base [20] for Israel.

### Sensitivity analyses

A number of sensitivity analyses have been performed to assess the robustness of the results of our study, where we varied; 1) inclusion of individual centres in the analysis (excluding the two centres with the most extreme results for effectiveness); 2) inclusion of patients in the analysis, according to the reason for ICU triage (ICU treatment/observation); 3) method used to estimate ICU daily costs.

#### Excluding centres with extreme results

Although in the main cost-effectiveness analyses we accounted for the variability across centres by use of multilevel models for effectiveness and cost estimates, it is possible that the overall results might have been driven by one or two centres with extreme results. In order to evaluate the robustness of our findings, we performed a sensitivity analyses where we excluded the two centres with the most extreme results, one suggesting a very large benefit of ICU admission (odds ratio of 0.3; 95% CI: 0.1 to 0.4) and one suggesting harm (1.2; 95% CI: 0.8 to 1.8), compared with the others.

#### Excluding problematic categories of patients

Although we adjusted the analyses for potential confounding factors and for study centre, still the presence of some categories of patients in our study population could have biased the results. Such patients include those referred to ICU only for observation and those admitted to ICU even if affected by terminal cancer. In both situations, the policy for ICU admission varies widely across centres, heavily depending on the availability of high dependency units or intermediate care units in the same hospital, as well as on cultural or religious factors. Therefore, we performed sensitivity analyses where we excluded: 1) patients referred to ICU for observation, thus only considering patients who had been triaged for ICU treatment; 2) patients with terminal cancer. Because direct information on the presence of terminal cancer was not available, we used a diagnosis of cancer accompanied with a Karnofsky score ≤50 (50 = "requires considerable assistance and frequent medical care") as a proxy for terminal cancer.

#### Estimating ICU daily cost based on level of care

In the ICU, unlike the ward, patients receive very different levels of nursing care. Nurses represent the largest cost component in ICU, so that different levels of nursing care translate into a high variability of daily cost per patient [1]. To account for differences in levels of nursing care, a sensitivity analysis based on a modified cost-block approach for the estimation of ICU daily cost per patient was performed, where the nursing cost was calculated based on information on daily specific procedures performed on the patient (see Additional file 1). Estimates of nursing cost per patient-day calculated using this method were entered into the cost block calculations in place of the average nurse cost derived from the annual nurse cost. This sensitivity analysis could only be performed on a subset of 37% of the patients admitted to ICU, for which information on daily procedures was available.

### Statistical analyses

Descriptive statistics utilised mean with standard deviation and median with interquartile range (IQR) as appropriate. Multilevel models were used for both effectiveness and cost analyses in order to account for the clustering effect induced by recruitment at multiple centres, namely cluster logistic regression and random effects linear regression, respectively. Selection of covariates to include in the regression models was performed based on adjusted R-square, using backward stepwise regression. The 95% confidence intervals for the estimates of the cost per life saved and cost per life-year saved were obtained using nonparametric bootstrap with replacement [21]. All analyses were performed using Stata 9.1 software (StataCorp. 2005. Stata Statistical Software: Release 9. College Station, TX, USA: StataCorp LP).

## Results

### Characteristics of the study population

A total of 7,449 patients were included in the study. Baseline characteristics for the whole study population and separately for patients accepted and not accepted in ICU are reported in Table 1. The two groups differed in terms of age, Karnofsky score and predicted mortality based on SAPSII, with patients not accepted to ICU being older, with worse chronic health and more severely ill. The analyses were, therefore, adjusted for these confounders. There was also variation in referral site, with rate of acceptance being much lower for emergency room and much higher for the recovery room/operating room. Similar differences were observed for type of referral, with a much higher acceptance rate for surgery compared with medical referrals. In the overall sample, 15% of the patients were refused admission, although ICU admission refusal rates varied widely across centres, (2%, 5%, 10%, 11%, 13%, 18%, 18%, 25%, 27%, 28%, 48%), partly due to differences in case-mix.

**Table 1:** Baseline characteristics of the study sample

Results		Overall population (*n *= 7,449)	Accepted (*n *= 6,312)	Rejected (*n *= 1,137)	*P-value*
Age					
	*Mean (SD)*	60.3 (18.0)	59.3 (18.0)	65.7 (17.3)	* < 0.001^a^*
	≥70 *(%)*	38	36	51	* < 0.001^b^*
Males *(%)*		58	58	57	*0.56 ^b^*
Karnofsky score					
	*Median (IQR)*	80 (70 to 90)	80 (70 to 90)	80 (60 to 90)	* < 0.001^c^*
	≤50*^d ^(%)*	14	12	24	* < 0.001^b^*
Predicted mortality (based on SAPS II)					
	* < 5 (%)*	29	30	20	* < 0.001^b^*
	*5 to 40 (%)*	54	52	60	
	* > 40 (%)*	18	17	20	
Referral site (%)					* < 0.001^b^*
	Operating/ Recovery room	34	39	5	
	A&E	29	26	47	
	Ward	28	25	46	
	Other hospital (excluding ICU)	7	8	1	
	Other ICU	2	2	1	
Type of referral (%)					
	Medical	52	46	86	* < 0.001^b^*
	Elective surgery	27	30	7	
	Emergency surgery	21	24	7	
Indication for referral (%)					
	ICU treatment				
	ICU	68	69	62	* < 0.001^b^*
	observation*^e^*	32	31	38	

*^a ^*t-test; *^b ^*chi-square test; *^c ^*Mann-Whitney test; *^d ^*from "Requires considerable assistance and frequent medical care" = 50 to "Dead" = 0 ("Normal no complaints; no evidence of disease" = 100); *^e^*Includes routine admission from theatre

LOS = length of stay.

### Effect of ICU admission on patient mortality

Results on length of stay (LOS) and mortality are reported in Table 2. Total hospital length of stay was higher in the accepted group (19.3 vs. 14.7; *P *< 0.001). For patients accepted to the ICU, the mean ICU LOS was 5.7 days (Standard Deviation 11.1), while the later ward LOS for these patients was 13.6 (23.6).

**Table 2:** Length of stay, mortality and cost

Results	Overall population (*n *= 7,449)	Accepted (*n *= 6,312)	Rejected (*n *= 1,137)	*P-value*
**Length of stay (LOS)**				
Total hospital LOS - days				
*Mean (SD)*	18.6 (28.0)	19.3 (27.0)	14.7 (32.7)	* < 0.001^a^*
**Mortality**				
28-day mortality *(%)*	24	22	33	* < 0.001^b^*
3-month mortality *(%)*	29	28	39	* < 0.001^b^*
**Cost**				
Cost per hospital stay ($)	13,443	14,851	5,629	* < 0.001^a^*
*Mean (SD)*	(20,780)	(21,597)	(12,947)	

*^a ^*t-test; *^b ^*chi-square test

The mortality at both 28 days and 3 months was significantly lower in the group admitted to the ICU. The results of the adjusted analyses of mortality are reported in Table 3. For mortality at 28 days, the estimate of the risk of death in accepted versus non-accepted patients, expressed in terms of odds ratio, was 0.7 (95% CI: 0.5 to 0.9; *P *= 0.017). The odds ratio increasingly favoured intensive care admission as predicted mortality rose. In patients with >40% predicted mortality the odds ratio reached 0.6 (95% CI: 0.4 to 0.8; *P *= 0.004).

**Table 3:** Results of the mortality analysis

		Predicted mortality		
Variable	ALL patients	< 5%	5% to 40%	> 40%
***Main analysis***				
Mortality at 28 days	0.7 (0.5 to 0.9)*	1.5 (0.8 to 2.8)	0.7 (0.5 to 1.0)*	0.6 (0.4 to 0.8)**
***Secondary analysis***				
Mortality at 3 months	0.7 (0.5 to 1.0)*	1.2 (0.6 to 2.3)	0.8 (0.6 to 1.0)	0.5 (0.3 to 0.8)**

Predicted mortality at the time of ICU triage based on SAPS II score. Reported are estimates of the risk of death in accepted versus non-accepted patients expressed in terms of Odds Ratio, with 95% Confidence Interval. *P < 0.05; **P < 0.01

### Costs

Total cost per hospital stay for patients accepted and not accepted into ICU are reported in Table 2. The mean daily cost per patient was $371 (€294) (95% CI: $368 (€292) to $374 (€296)) for the ward stay and $1,339 (€1,063) (95% CI: $1,334 (€1,059) to $1,343 (€1,066)) for the ICU stay.

After adjusting the analyses of costs for the same variables, the estimated difference in costs per patient between accepted and not accepted was $6,156 (€4,886) (95% CI: $5,028 (€3,990) to $7,283 (€5,780)).

### Cost effectiveness of ICU admission

Based on the results of the adjusted analyses of 28-day mortality and costs, the estimate of cost per life saved was $103,771 (€82,358) (95% CI: $56,855 (€45,123) to $150,687 (€119,593)). The values of life expectancy assigned to each patient gave an average life expectancy in our population of 14.7 years after hospital discharge. Using this average figure, a cost per life-year saved of $7,065 (€5,607) (95% CI: $3,871 (€3,072) to $10,259 (€8,142)) was obtained. The cost effectiveness of ICU admission increased with increasing predicted mortality (Table 4).

**Table 4:** Results of the cost-effectiveness analysis

		Predicted mortality		
Cost-effectiveness analysis	ALL patients	< 5%	5% to 40%	> 40%
Cost per life saved	103,771 (56,855 to 150,687)	-197,195 (-3,635,418 to 3,241,028)	117,675 (36,241 to 199,109)	60,046 (24,521 to 95,571)
Cost per life-year saved	7,065 (3,871 to 10,259)	-13,426 (-247,510 to 220,658)	8,012 (2,467 to 13,556)	4,088 (1,669 to 6,507)

Estimates of mortality adjusted as in Table 3, and estimates of costs adjusted for the same variables. Reported are costs in $, with 95% CI

Estimates of cost per life saved and cost per life-year saved were similar when considering mortality at three months, $103,418 (€82,078) (95% CI: $44,198 (€35,078) to $162,639 (€129,079)) and $7,041 (€5,588) (95% CI: $3,009 (€2,388) to $11,073 (€8,788)), respectively.

### Sensitivity analyses

#### Excluding centres with extreme results

In this sensitivity analysis we excluded 1,471 patients (19.7% of the whole sample) from two centres, which showed the most extreme positive and negative mortality results. Results of the analysis for 28-day mortality were a cost per life saved of $119,301 (€94,683) ($26,581 (€21,096) to $212,020 (€168,270)) and a cost per life-year saved of $8,121(€6,445) ($1,810 (€1,437) to $14,432 (€11,454)).

#### Excluding problematic categories of patients

When excluding patients referred to ICU for observation (*n *= 2,363; 32% of the whole sample), 13.9% of the patients were refused admission. The results for the effect of ICU admission on mortality at 28 days (0.7; 95% CI: 0.5 to 0.9; *P *= 0.020) were the same as those in the main analysis, while the difference in costs between admitted and non-admitted patients was higher at $10,409 (€8,261) ($8,479 (€6,729) to $12,340 (€9,794); *P *< 0.001). As a consequence, this sensitivity analysis suggests a lower cost effectiveness of ICU admission compared with the main analysis, although the estimates of the two cost-effectiveness measures became less accurate due to the loss of nearly one third of the sample size (cost per life saved: $142,806 (€113,338); $57,233 (€45,423) to $228,378 (€181,252); cost per life-year saved: $9,721 (€7,715); $3,895 (€3,091) to $15,546 (€12,338)).

Results of the sensitivity analysis with exclusion of terminal cancer patients, defined as patients with diagnosis of cancer and Karnofsky ≤50 (*n *= 220; 3% of the sample), were substantially the same as those of the main analysis: cost per life saved: $108,257 (€85,918) ($51,139 (€40,587) to $165,375 (€131,250)); cost per life-year saved: $7,370 (€5,849) ($3,482 (€2,763) to $11,259 (€8,936)).

#### Estimating ICU daily cost based on level of care

If the costing method for ICU was modified to account for the different levels of care received by patients accepted into ICU, the cost per life saved was $94,898 (€75,316) (95% CI: $24,570 (€19,500) to $165,226 (€131,132)) with a cost per life-year saved of $6,461 (€5,128) (95% CI: $1,673 (€1,328) to $11,249 (€8,928)). So the different levels of care had minimal effect upon the costs.

In all sensitivity analyses, similar results were obtained when considering three-month mortality. The same pattern of cost effectiveness increasing with increasing predicted mortality was also observed.

## Discussion

The widely held view of most health care professionals is that intensive care is a high cost specialty with demand exceeding supply. This has effectively led to rationing [22-27]. An exploratory study in the UK in 1997 examined the mortality among referred patients who were refused admission [28]. This study concluded that there was a higher rate of attributable mortality in patients not admitted, but the authors were not persuaded that the solution was to have more beds but rather clearer guidelines on appropriate admission and discharge criteria. Studies have found that refusal of admission to the ICU is common [29], ranging from 38% [22] to 24%, [5]. This may have led to undertreatment or under admission of patients, as illustrated in chronic obstructive pulmonary disease patients by Wildman [30]. In a large proportion the patients who were not admitted had more severe acute illness, as reflected by a higher Acute Physiology and Chronic Health Evaluation II (APACHE II) score [22]. However, the frequency of admission decreased when the ICU was full [5] despite the fact that admission to the ICU was associated with a lower mortality.

One of the earliest studies to measure cost effectiveness in the ICU evaluated 211 patient stays in hospitals in the Paris region [9]. It calculated cost per ICU stay, cost per life saved and cost per quality adjusted life-year (QALY) saved. This study concluded that the cost per life-year saved was $1,150 (€913) and cost per QALY was $4,100 (€3,254) in 1996. A further prospective study of 303 consecutive medical ICU patients reported a cost per life-year saved of 19,330 € ($28,354) [10] in 1998. However, in both studies patients not admitted were not investigated and the assumption made that a "do nothing" strategy had a theoretical certainty of death. Although patients refused admission are more likely to die, the assumption that a refused admission means certain death is not sustainable. Patients may be refused admission on the grounds that treatment is futile or that they are currently not ill enough to benefit from ICU therapy more than conventional ward treatments. For example, in one study [5] the mortality rate for patients not admitted was 46% and in the other study [22] the standardised mortality ratio (SMR) for those admitted was 0.93, whereas the SMR for those triaged and then refused admission was 1.76. The present study differed from the previous studies [7-10] in many ways. The cost effectiveness of admission to intensive care after ICU triage was assessed by comparing ICU care with the alternative; that is, ward care. Thus, mortality and cost of the 6,312 patients admitted to ICU were compared with those of the 1,347 patients not admitted to ICU. The number of patients in the present study was more than 20 times larger, and both medical and surgical ICU admissions were included in 11 ICUs in 7 different European countries, all with different admission policies. While not representative of individual countries, the present results encompass a wide sample. In addition, the study adjusted for confounding factors such as age and chronic health status, and the results were stratified by the severity of acute illness.

In the overall population, the cost per life saved was $103,771 (€82,358), while the cost per life-year saved was $7,065 (€5,607), with an average predicted life expectancy of close to 15 years. These costs fall as predicted mortality rises, suggesting that intensive care becomes more cost effective for patients who are referred to ICU with greater severity of illness. For patients with more than 40% chance of death, cost per life saved is about $60,000 (€47,619) and per life-year saved $4,000 (€3,175).

The cost per life-year saved was derived using an average figure for life expectancy of about 15 years, which was based on study individuals' age, sex, country of origin (data from the general population) and adjusted for the initial excess mortality of ICU survivors. This "excess mortality" was assumed to apply to all the patients referred for ICU admission, regardless of whether they were accepted or not. This model assumes that the excess mortality is associated with the critical illness, rather than due to adverse effects of the additional treatments given on ICU. It is important to realise that the cost-effectiveness estimates reported in this paper are only valid "on average", and thus these figures are not aimed at changing the clinical logic of admitting or rejecting individual patients. There will always be a vital clinical decision to make regarding whether a patient can benefit appropriately from critical care. This will be highly influenced by the likely survival for the critical illness itself, the underlying acute illness, chronic pre-existing medical conditions and other longer term illness or quality of life factors. Probably the most important aspect of these results is that they have the potential to influence the overall funding provision for critical care facilities and staff.

The figures of cost effectiveness of ICU admission reported in this study are comparable with or, in some cases, compare favourably with, those of other interventions. For example, the cost per life-year saved by screening for breast cancer has been reported as £8,561 (€6,794) in 1996, equivalent to about $12,235 [31], and that of cervical cancer screening £36,000 (€28,571) per life saved (reported in 2004) equivalent to $51,450 [32]. The cost per life-year saved of mechanical ventilation used for life threatening stroke has been reported at $37,600 (€29,841) [33] in 1996; whereas, the cost per life-year saved of implantable cardioverter-defibrillators was $235,000 (€186,508) [34] (reported in 2006). Consequently, ICU treatment seems to be as cost effective as some other interventions whose cost effectiveness is more generally accepted. However, our study suggests that ICU admission of patients with very low predicted mortality (for example, less than 5%) may be inappropriate both clinically and economically. It could be argued that increased critical care provision would lead to a higher number of admissions with a very low predicted mortality, which might therefore decrease cost effectiveness. However, there are reasons to suspect that this might not be the case. The converse could be true; that it would lead to an increase in suitable admissions (perhaps by an increased throughput of major surgery in patients with some comorbidities) or even a more rapid admission rate of current patients, so enabling the quicker introduction of appropriate life saving therapies, some of which are complex and some of which are relatively simple but are often delayed on the ordinary ward. This could potentially lead to an improvement in outcome.

As with every study there are limitations. It could be argued that a more thorough and consistent study design would have been to standardise the treatment received on the ward and on the ICU to ensure that any inconsistent absence of certain therapies, facilities or techniques on the ward or the ICU would not have impacted on survival. However, in the critically ill there are research ethic difficulties with not assessing the suitability of each patient for a particular type of treatment or level of escalation of therapy, whether using ward based or ICU based care. The practicalities of implementing a standard regime on all sites and in all groups would have been exceptionally difficult, so reducing the numbers of possible centres or patients even if ethical issues could be overcome. Therefore, a simpler design was adopted, that would also avoid the problems of many subjects refusing to take part, which would have added its own selection bias.

Because we did not have direct information on terminal cancer, we used as a proxy a diagnosis of cancer together with a poor Karnofsky score. We then performed a sensitivity analysis with exclusion of these patients, which showed results very similar to those of the main analysis. Although the use of this proxy may have caused the wrong inclusion of some cancer patients who were not terminal but had low Karnofsky scores due to other severe comorbidities, this sensitivity analysis would have detected substantial bias if there were any.

The major limitation of this study is inherent to its observational nature, where the decision to accept a patient into ICU or not is influenced by factors associated with the outcome of the patient. Although we adjusted the analyses for important confounding factors, including measures of severity of illness, residual confounding is likely to be present. Moreover, we used a top-down approach to the costing of ICU and ward, while ideally the costing method should be bottom up (microcosting), where the individual items of cost are accounted for, and totalled. This method, however, is too expensive to be currently viable throughout Europe. The fact that the use of an alternative and more sensitive approach to costing the ICU stay, based on the level of care received by the patient, did not modify our results suggests robustness of the findings. It is also reassuring to see that our estimate of an average daily ICU cost in the 11 centres using a top-down approach of €1,028 is indeed close to the figure of €923 found in 51 ICUs in German teaching hospitals obtained by Moerer *et al. *[35] through the use of a bottom-up approach.

## Conclusions

It has been widely assumed that ICU care is expensive and this has almost certainly encouraged an unreasonably low level of provision of ICU beds and resources. The average cost per life saved of $103,771 (€82,358) and an average cost per life-year gained of $7,065 (€5,607) observed in our study would suggest that this assumption is incorrect. This information is important for health care providers who need to balance the costs and benefits of ICU against those of other types of health care. Our results may also influence those who sanction the construction of critical care facilities in the future and may allow those who manage critical care to argue more effectively that critical care potentially does represent value for money and should have a similar priority to other therapies where a higher level of financial provision is regarded as the norm.

## Key messages

• The daily cost of intensive care has been described, but randomised controlled studies to assess cost effectiveness of admission to intensive care are unlikely to be feasible.

• Observational studies looking at the cost effectiveness of admission to intensive care after ICU triage have generally assumed that the result of non-admission would be death, but some patients do survive.

• This observational study attempts to determine cost effectiveness for intensive care admission by observing patients who are admitted or refused admission to intensive care.

• The findings of this study suggest that ICU admission is cost effective, with cost per life saved and per life-year saved comparable to, or less than, established therapies used elsewhere in medicine.

## Abbreviations

APACHE II: Acute Physiology and Chronic Health Evaluation II; ARR: absolute risk reduction; CER: control event rate; CI: confidence interval; CLS: cost per life saved; CLYS: cost per life-year saved; ELDICUS: triage decision-making for the "Elderly in European Intensive Care Units"; HLC: high level care; ICER: incremental cost effectiveness ratio; ILC: intermediate level care; IQR: interquartile range; LE: life expectancy; LLC: low level care; OR: odds ratio; PPP: Purchasing Power Parity; QALY: quality adjusted life year; SAPS II: Simplified Acute Physiology Score II.

## Competing interests

The authors declare that they have no competing interests.

## Authors' contributions

DLE and CLS conceived the study and applied for grant funding. CLS led the overall grant project. CM led the statistical analysis. DLE, GHM and GI assisted with the analysis and design of the paper. GHM led the writing of the paper and led MERCS, the data collating centre. GHM, DLE, CM and GI helped to draft the initial manuscript. DC provided statistical advice. PJ advised on health economics. Most authors, including DLE, GHM, GI, APez, AL, JW, RP, APes, NP, DP, GG, JB, JK, CH, SLC, MB, AA and CLS, either led the organisation and data gathering in one of the centres or had a major role in the coordination of the centres. All authors have read and approved the manuscript.

## Supplementary Material

Additional file 1 **Supplementary material**. Details on methods used to estimate cost per life saved, cost per life-year saved, and ICU daily cost per patient based on level of care. Click here for file
