# Supplementary material for: Implications of ICU triage decisions on patient mortality: a cost-effectiveness analysis
Source: Crit Care. 2011 Feb 9;15(1):R56. doi: 10.1186/cc10029 (PMC3221989; doi:10.1186/cc10029)
Supplement: Additional file 1 — Supplementary material. Details on methods used to estimate cost per life saved, cost per life-year saved, and ICU daily cost per patient based on level of care. [file cc10029-S1.DOC]

**SUPPLEMENTARY MATERIAL**

*Cost per life saved*

The cost per life saved, or Incremental Cost-Effectiveness Ratio (ICER), is the difference in cost divided by the difference in mortality rates (Absolute Risk Reduction, ARR). The ARR was estimated from the odds ratio (OR) derived from the adjusted analyses for mortality, as follows:

ARR = ((1-CER)*(CER)*(1-OR)) / (1-(CER*(1-OR)))

where the control event rate (CER) corresponds to the mortality in patients not admitted to ICU.

*Cost per life-year saved*

Life expectancy for hospital survivors was estimated by accounting for the excess mortality that ICU patients experience in the first four years after hospital discharge compared with the general population. We estimated life expectancy in the same way for patients whose admission to ICU was rejected, on the basis that they were likely to be affected by similarly severe disease and thus experience similar excess mortality as patients accepted into ICU. The cumulative figures for excess mortality were derived from Ridley and colleague [36] and were as follows; 1^st^ year: 18%; 2^nd^ year: 23%; 3^rd^ year: 29%; 4^th^ year: 32%. After the first four years, life expectancy of survivors was assumed to be back to that of the general population [36], and a figure of life expectancy was assigned to each patient based on their age at hospital discharge, gender and country of residence. Data on life expectancy in the general population were obtained from EUROSTAT [19] for European countries, and from U.S Census Bureau, International Data Base [20] for Israel.

Cost per life-year saved (CLYS) was then calculated based on the cost per life saved (CLS) and life expectancy (LE) as follow:

CLYS = CLS/mean LE

*ICU daily cost per patient based on level of care*

To allow an assessment of the intensity of nursing care, additional data was collected on a subset of consecutive patients accepted in ICU during the first six months of the study in 10 out of the 11 participating hospitals (37% of all accepted ICU patients). The information was collected daily on specific aspects of patient’s care in order to classify each ICU day as high, intermediate or low nursing level of care (HLC, ILC and LLC, respectively) [37]. Each day in the ICU was defined as; HLC in the presence of mechanical ventilation *or* multiple vasoactive medications; ILC in the presence of at least two of the following: supplementary ventilatory care, single vasoactive medication, any dialysis/ultrafiltration procedure; LLC in any other case. The nursing cost for each ICU day for a specific patient was then calculated by defining a nurse to patient ratio corresponding to the observed level of care. Nurse to patient ratios were defined differently across centres, depending on the nursing provision available in that institution for different levels of care. In centres which had 3.5 nurses per bed or more, the nurse to patient ratio was defined as 1:1, 1:2 and 1:3 for HLC, ILC and LLC, respectively, while in centres with fewer than 3.5 nurses per bed, the corresponding figures were 1:2, 1:3 and 1:4. Estimates of nursing cost per patient-day calculated using this method were entered into the cost block calculations instead of the average nurse cost.
